# A healthy dietary metabolic signature is associated with a lower risk for type 2 diabetes and coronary artery disease

**DOI:** 10.1186/s12916-022-02326-z

**Published:** 2022-04-21

**Authors:** Einar Smith, Ulrika Ericson, Sophie Hellstrand, Marju Orho-Melander, Peter M. Nilsson, Céline Fernandez, Olle Melander, Filip Ottosson

**Affiliations:** 1 Department of Clinical Sciences, Lund University, Jan Waldenströms gata 35, 91-12-027, SE-214 28 Malmö, Sweden; 2 Department of Emergency and Internal Medicine, Skåne University Hospital, Malmö, Sweden; 3 Section for Clinical Mass Spectrometry, Danish Center for Neonatal Screening, Department of Congenital Disorders, Statens Serum Institut, Copenhagen, Denmark

**Keywords:** Metabolomics, Nutrition, Dietary biomarker, Type 2 diabetes, Coronary artery disease

## Abstract

**Background:**

The global burden of cardiovascular disease and type 2 diabetes could be decreased by improving dietary factors, but identification of groups suitable for interventional approaches can be difficult. Reporting of dietary intake is prone to errors, and measuring of metabolites has shown promise in determining habitual dietary intake. Our aim is to create a metabolic signature that is associated with healthy eating and test if it associates with type 2 diabetes and coronary artery disease risk.

**Methods:**

Using plasma metabolite data consisting of 111 metabolites, partial least square (PLS) regression was used to identify a metabolic signature associated with a health conscious food pattern in the Malmö Offspring Study (MOS, *n* = 1538). The metabolic signature’s association with dietary intake was validated in the Malmö Diet and Cancer study (MDC, *n* = 2521). The associations between the diet-associated metabolic signature and incident type 2 diabetes and coronary artery disease (CAD) were tested using Cox regression in MDC and logistic regression in Malmö Preventive Project (MPP, *n* = 1083). Modelling was conducted unadjusted (model 1), adjusted for potential confounders (model 2) and additionally for potential mediators (model 3).

**Results:**

The metabolic signature was associated with lower risk for type 2 diabetes in both MDC (hazard ratio: 0.58, 95% CI 0.52–0.66, per 1 SD increment of the metabolic signature) and MPP (odds ratio: 0.54, 95% CI 0.44–0.65 per 1 SD increment of the metabolic signature) in model 2. The results were attenuated but remained significant in model 3 in both MDC (hazard ratio 0.73, 95% CI 0.63–0.83) and MPP (odds ratio 0.70, 95% CI 0.55–0.88). The diet-associated metabolic signature was also inversely associated with lower risk of CAD in both MDC and MPP in model 1, but the association was non-significant in model 3.

**Conclusions:**

In this proof-of-concept study, we identified a healthy diet-associated metabolic signature, which was inversely associated with future risk for type 2 diabetes and coronary artery disease in two different cohorts. The association with diabetes was independent of traditional risk factors and might illustrate an effect of health conscious dietary intake on cardiometabolic health.

**Supplementary Information:**

The online version contains supplementary material available at 10.1186/s12916-022-02326-z.

## Background

Cardiovascular disease is the leading cause of death worldwide and 1 in every 11 adults has type 2 diabetes globally which has detrimental effect on quality of life and life expectancy among millions of people annually [1, 2]. There is increasing evidence that many cases of type 2 diabetes and cardiovascular disease could be prevented by maintaining a healthy diet [2, 3]. The current evidence is suggesting that a healthy diet is low in red meat, processed meat, refined grains and sugar-sweetened beverages and rich in whole grains, fruits, vegetables, nuts and legumes [2, 3].

The interaction between health and dietary intake is complex. Recently, the use of dietary patterns has become increasingly common in nutritional epidemiology as a tool to distinguish between different dietary habits and to assess the health effect of food intake [4, 5]. Several data-driven methods have shown adherence to health-conscious dietary patterns to be associated with a lower risk for type 2 diabetes and cardiovascular disease [4–6]. The health conscious/prudent dietary patterns often have a similar composition as the diets that are currently being recommended for type 2 diabetes and cardiovascular disease prevention [4–6].

The use of metabolite measurement in nutrition research is common as food or food component intake biomarkers provide an objective assessment of dietary intake unaffected by the inherent difficulties of dietary intake reporting [7, 8]. We have previously investigated the relationship between a data-driven healthy dietary pattern and single metabolites and found several dietary pattern associated metabolites to be associated with future risk for cardiovascular disease [9].

Metabolite patterns rather than single metabolites might better reflect the overall adherence to dietary patterns [7, 10]. Dietary pattern associated biomarkers also have the potential to not only capture the intake of nutrients, but also individual variation in the microbiota- and endogenous metabolism of food [11]. Several studies have shown promising results using multivariate metabolomics modelling to discriminate participants based on their dietary intake [12–14].

Our aim in this study is two-fold. First, we seek to use multivariate methodology to create a metabolic signature of a health conscious dietary pattern and test its validity in different cohorts. We will then investigate the association of the diet associated metabolic signature with coronary artery disease and type 2 diabetes in two separate cohorts. This approach may pave the way for the identification of individuals with unhealthy eating habits and a higher risk for cardiovascular disease and type 2 diabetes with a single plasma sample.

## Methods

### Cohort descriptions

We conducted this study using data from three different Swedish cohorts: the Malmö Offspring Study (MOS) [15], the Malmö Diet and Cancer study (MDC) [16] and the Malmö Preventive Project (MPP) [17]. As described below, a diet associated metabolic signature was generated and internally validated in MOS and further externally validated in MDC. The associations with the metabolic signature and future type 2 diabetes and CAD were tested in both MDC and MPP.

MOS is an ongoing cohort study that was launched in 2013 to map risk factors for chronic diseases [15]. In our study, the study sample consisted of 1538 individuals with overlapping data on metabolomics and adherence to a previously derived data-driven healthy food pattern [18].

MDC is a population-based prospective cohort study consisting of 28 098 individuals who attended baseline examination between 1991 and 1996 [16]. We had previously included a random sample of 3833 participants from the MDC cardiovascular cohort [9], and out of these, 2684 had information on adherence to a previously derived data-driven healthy food pattern [6]. After exclusion of participants with prevalent coronary artery disease (CAD) (*n* = 0) or prevalent type 1 or type 2 diabetes (*n* = 138), missing data on alcohol intake (*n* = 1) or smoking status (*n* = 7), or unknown vital status due to emigration (*n* = 17), 2521 individuals remained and were used in the statistical analyses.

MPP is another population-based prospective cohort, with 33,346 individuals enrolled between 1974 and 1992. Between 2002 and 2006, all participants still alive were invited to a re-examination, which serves as baseline in this study. Among a random sample of 5386 individuals out of the 18,240 that attended re-examination, we have previously created a nested case-control study design [17]. Among the 5386 individuals, 1406 were excluded due to prevalent type 2 diabetes, CAD or because of incomplete data on CAD risk factors or missing plasma samples. Out of the remaining 3980 individuals, 382 developed CAD before December 31, 2013, and 203 developed type 2 diabetes. In total, 35 individuals developed both type 2 diabetes and CAD. The remaining 3361 individuals qualified as controls due to them not developing CAD or type 2 diabetes during follow-up. Due to high analytical demand, 498 were randomly included in the analyses as controls, resulting in a baseline study sample of 1083 individuals. The median follow-up time for type 2 diabetes was 6.3 years and for CAD 7.2 years.

### Covariate collection

At the baseline examination of respective cohort, covariate collection was done primarily through questionnaires combined with a visit to a research nurse whom conducted standardised anthropometrics analyses and blood sampling. BMI was calculated using the weight and height measured at the baseline visit. Supine blood pressure (mm Hg) was measured once after 10 min rest. The usage of anti-hypertensive medicine was identified through a questionnaire where participants listed their daily medications.

In MDC, physical activity was assessed using a questionnaire including 17 different activities adapted from the Minnesota Leisure Time Physical Activity Questionnaire and split into three equally big groups: low, medium and high [6]. In MPP, physical activity was classified according to four different categories in a questionnaire as previously described [19]. The highest group had only two participants so they were moved into the “high” activity group so that three groups remained. Participants with missing data on physical activity were imputed into the largest middle group. Smoking status was defined as smoking or non-smoking using self-reporting. Ex-smokers were defined as non-smokers. The total consumption of alcohol was in MDC defined by a four-category variable created by combining information from the questionnaire and the 7 day menu book as previously described [6]. After the above described exclusion in MDC, combined with the imputation of physical activity in MPP, there were no missing values for the covariates.

Baseline blood samples were drawn for analysis of blood lipids (total and HDL-cholesterol and triglycerides) and blood glucose according to standard procedures at the Department of Clinical Chemistry, Malmö University Hospital. LDL-cholesterol concentration was calculated according to Friedewald formula. An aliquot of plasma samples were collected in citrate-coated vials in MDC and EDTA-coated vials in MPP and MOS and frozen to − 80° until extraction for metabolomics analysis as described below.

### Follow-up data

Endpoints were retrieved by linking the ten digit Swedish personal identification number with three registers: the Swedish Hospital Discharge Register, the Swedish Cause of Death Register, and the Swedish Coronary Angiography and Angioplasty Registry (SCAAR) as previously described [9]. These registers have been previously described and validated for classifications of outcomes [20]. CAD was defined as coronary artery revascularization, fatal or non-fatal myocardial infarction or death due to ischemic heart disease. Myocardial infarction was defined on the basis of the International Classification of Diseases (ICD) 9 code 410 or ICD-10 code I21. Death attributable to ischemic heart disease was defined as ICD-9 codes 412 and 414, or ICD-10 codes I22, I23, or I25. Coronary artery bypass surgery was identified from the national Swedish classification systems of surgical procedures and defined as procedure codes 3065, 3066, 3068, 3080, 3092, 3105, 3127, or 3158 in the Op6 system or as procedure code FN in the KKÅ97 system. Percutaneous coronary intervention was identified from SCAAR [21].

Incident diabetes cases were retrieved from six different national and regional diabetes registers as described elsewhere [22]. Prevalent diabetes mellitus at baseline was defined as a fasting whole blood glucose ≥ 6.1 mmol/L (corresponding to a plasma glucose of ≥ 7.0 mmol/L) or a history of physician diagnosis of diabetes mellitus or being on antidiabetic medication or having been registered in any of the six different national and regional diabetes registers.

The date of last follow-up was 2016-12-31 in MDC and 2013-12-31 for MPP.

### Health conscious food patterns

In this study, we utilised two published data-driven dietary patterns, a health-conscious food pattern from MOS [18] and a health-conscious food pattern from MDC [6] which both were created using principal component analysis to reduce food groups to dietary patterns. In MDC, the dietary data was collected using a modified diet history method that combined a 7-day menu book, a food frequency questionnaire and a 45-min interview [23, 24]. In MOS, the diet was assessed using the 4-day online food record Riksmaten2010, developed by the Swedish National Food Agency and a short food frequency questionnaire [25, 26]. The food patterns consisted of similar loadings in MOS and MDC (Additional file 1: Supplementary method).

### Metabolomics analysis

Profiling of plasma metabolites was performed using LC-MS using a UPLC-QTOF-MS System (Agilent Technologies 1290 LC, 6550 MS, Santa Clara, CA, USA) and has been described elsewhere [27]. Briefly, over-night fasted plasma samples were extracted and subsequently separated on an Acquity UPLC BEH Amide column (1.7 μm, 2.1 × 100 mm; Waters Corporation, Milford, MA, USA).

We identified metabolites by matching the measured mass-over charge ratio (m/z) and chromatographic retention times with an in-house metabolite library consisting of 111 metabolites that were measurable on all three cohorts (Additional file 1: Table S1). Out of 111 metabolites, 25 of them, mostly consisting of acylcarnitines had putative identities based on their fragmentation spectra and the rest had confirmed identities (Additional file 1: Table S1). Metabolite peak areas were integrated using Agilent Profinder B.06.00 (Agilent Technologies, Santa Clara, CA, USA). The normalisation process of metabolite levels is described in the supplementary method (Additional file 1: Supplementary method) [28].

### Statistical analyses

All statistical analyses were done using R (version 4.0.4). To create a metabolic signature for health-conscious eating in MOS, partial least square (PLS) regression was applied with metabolite data as X and the health-conscious food pattern in MOS as Y using the package mixOmics (version 6.14.0) [29]. The model was trained in 80% randomly selected participants from MOS. The number of principal components included in the model was determined by calculating the Q2 (predicted variation) and R2 (explained variation) values using ten-fold cross validation and a threshold of Q2 > 0.0975 [30]. This resulted in only one principal component, named the metabolic signature. The results were validated in the remaining 20% using Pearson correlation after calculating the metabolic signature using the “Predict” function in mixOmics. We tested correlations between the metabolic signature and intake of food groups in MOS with Pearson correlation. The “Predict” function in mixOmics was further used to calculate the metabolic signature in MDC and MPP. The correlation between the metabolic signature and the health-conscious food pattern in MDC was tested using Pearson correlation as well as partial Pearson correlation adjusted for sex, age and body mass index (BMI).

To test the associations between the metabolic signature and type 2 diabetes and CAD, together referred to as cardiometabolic disease, prospective data was used in both MPP and MDC. First, we constructed Kaplan–Meier curves in MDC for type 2 diabetes and CAD separately with participants split into quintiles of the metabolic signature. Differences in risk in the Kaplan–Meier analysis between quintiles were evaluated using the log rank test.

To further explore the phenotype of the metabolic signature, baseline characteristics were summarised by quintile of the metabolic signature in both MPP and MDC. The differences were tested using ANOVA for continuous variables and chi-square test for categorical variables.

For the remainder of the logistic and proportional hazard regression analyses, the metabolic signature was added as a mean centred and unit variance scaled continuous variable.

In MDC, Cox proportional hazards regression was used to create three models associating the metabolic signature with CAD and type 2 diabetes separately. Model 1 was unadjusted; model 2 was adjusted for the potential confounders smoking, age, sex, alcohol intake and physical activity. Model 3 was additionally adjusted for the potential mediators LDL cholesterol, HDL cholesterol, glucose, triglycerides, BMI, systolic blood pressure and treatment of anti-hypertensive medicine. Model 2 was to be considered the main analyses while model 3 further included adjustments for the above-mentioned potential mediators as previously known risk factors for cardiometabolic disease. Smoking status, sex, alcohol intake and physical activity were adjusted for as categorical variables and the remaining covariates were adjusted for as continuous variables. The proportional hazard assumption was tested using the “coxzph” function in the “Survival” package [31]. Years to event or to last follow-up was used as the underlying time variable in the Cox regressions. The association between the metabolic signature and CAD in MDC was also tested with logistic regression. As MPP had a nested case-control design as previously described, we used logistic regressions to test the association between the metabolic signature and future disease. We created three models for CAD and three models for type 2 diabetes that were adjusted for the same variables as Cox regression models 1-3 except for alcohol intake, which was not included in the MPP models as MPP has no baseline estimate of alcohol intake. Analyses were considered significant if the *p* value was below 0.05.

## Results

The baseline characteristics of the study participants in MOS, MDC and MPP can be found in (Table 1). The participants in MPP were older and had a higher proportion of men than in MDC and MOS and had higher fasting glucose and BMI. Out of the 2521 participants in the MDC cohort, 322 participants developed type 2 diabetes and 303 CAD during a median follow-up time of 25.1 years.

**Table 1:** Baseline study characteristics

	Malmö Offspring Study (MOS) mean (SD) or %	Malmö Diet and Cancer (MDC) mean (SD) or %	Malmö Preventive Project (MPP) mean (SD) or %
Participants (*n*)	1538	2521	880/701^a^
Dietary data	Yes	Yes	No
Age (years)	40.4 (13.9)	57.4 (6.0)	69.5 (6.1)
Sex (% female)	54.2%	59.5%	28.6%
LDL (mmol L^−1^)	3.13 (0.93)	4.13 (0.96)	3.78 (0.98)
HDL (mmol L^−1^)	1.64 (0.48)	1.42 (0.37)	1.36 (0.41)
TG (mmol L^−1^)	1.09 (0.67)	1.23 (0.57)	1.24 (0.63)
Glucose (mmol L^−1^)	5.49 (1.00)	4.90 (0.43)	5.55 (0.59)
BMI (kg m^−2^)	25.6 (4.5)	25.3 (3.7)	27.1 (4.3)
Systolic blood pressure (mm Hg)	117.6 (15.6)	140.6 (18.5)	146.4 (21.5)
Current smoker	32.8%	28.1%	21.4%
Anti-hypertensive treatment	8.7%	11.3%	39.9%

Values are displayed as mean (SD) or percentages

*BMI* Body mass index, *LDL* LDL cholesterol, *HDL* HDL cholesterol, *TG* Triglycerides, *CAD* Coronary artery disease

^a^The MPP cohorts had 880 participants included in the CAD nested case control study and 701 included in the type 2 diabetes nested case control study

**Fig. 1:**
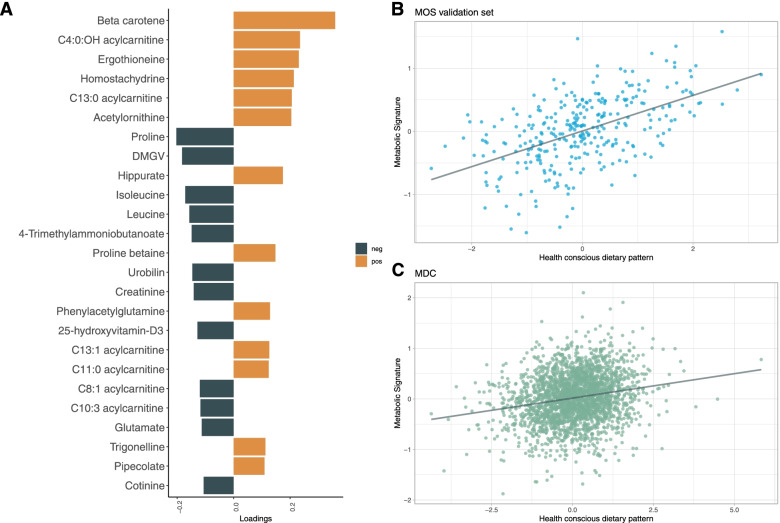
Metabolic signature model. **A** The 25 metabolites with the strongest influence on the component in the metabolic signature. **B** Association with the metabolic signature and health-conscious food pattern in the validation cohort in MOS. **C** Association with the metabolic signature and the health conscious food pattern in MDC. DMGV, dimethylguanidino valerate

We created a metabolic signature for the health-conscious food pattern trained on metabolite data in 80% of participants in MOS using partial least square regression. Using tenfold cross-validation resulted in a model with one component, as the second component had a Q2 (predicted variation) value of 0.076, which was lower than the predefined cut-off 0.0975. This indicates that the predictive power does not increase by using 2 components instead of 1. The single retained component had a moderate Q2 value of 0.29 and a moderate R2 (explained variation) value of 0.28 in the training set in MOS. The unadjusted correlation between the metabolic signature and the health conscious dietary pattern was strong in the validation subset of MOS (*ρ* = 0.52, 95% CI 0.44–0.60, *p* = < 0.0001) (Fig. 1B). The R2 in the validation subset was 0.27. The metabolite beta carotene contributed the most to the model component as positive loading followed by C4:OH-acylcarnitine, ergothioneine, homostachydrine, C13:0-acylcarnitine and acetylornithine (Fig. 1A). The metabolites that contributed the most as negative loadings in the model component were proline, dimethylguanidino valerate (DMGV) and isoleucine (Fig. 1A). The complete model loadings can be found in the supplementary material (Additional file 1: Table S1).

The metabolic signature correlated with food groups that contributed to the loadings in the health-conscious food pattern in MOS (Additional file 1: Fig. S1). The largest correlations were with fruit and berries (*ρ* = 0.34), non-legume-vegetables (*ρ* = 0.25), tea (*ρ* = 0.23), legumes (*ρ* = 0.20) and nuts and seeds (*ρ* = 0.18). The largest negative correlations were with low fibre bread (*ρ* = − 0.28), sugar sweetened beverages (*ρ* = − 0.26), red non-processed meat (*ρ* = − 0.25) and processed meat (*ρ* = − 0.19).

The metabolic signature trained in MOS was used to extrapolate a metabolic signature in MDC and MPP using metabolite levels. The predicted metabolic signature correlated moderately with the health-conscious dietary pattern in MDC (*ρ* = 0.20, 95% CI 0.16–0.24, *p* = < 0.0001) (Fig. 1C). Adjusting the correlation model for BMI, sex and age did not affect the correlation coefficient (*ρ* = 0.20, 95% CI 0.18–0.22, *p* = < 0.0001). In MDC, MPP and MOS respectively, individuals in quartile 1 of the metabolic signature were more predominantly male, had lower fasting HDL cholesterol, higher fasting glucose higher BMI and higher systolic blood pressure (Additional file 1: Tables S2-S4). The mean BMI of participants in MDC of quintile 1 of the metabolic signature was 26.8 compared to 23.8 in quintile 5. In MPP, quintile 1 of the metabolic signature had a mean BMI of 29.0 compared to the mean BMI of 25.0 in quintile 5. The numerically greatest attenuation by “one-by-one” risk factor adjustments stemmed from BMI (Additional file 1: Table S5).

**Fig. 2:**
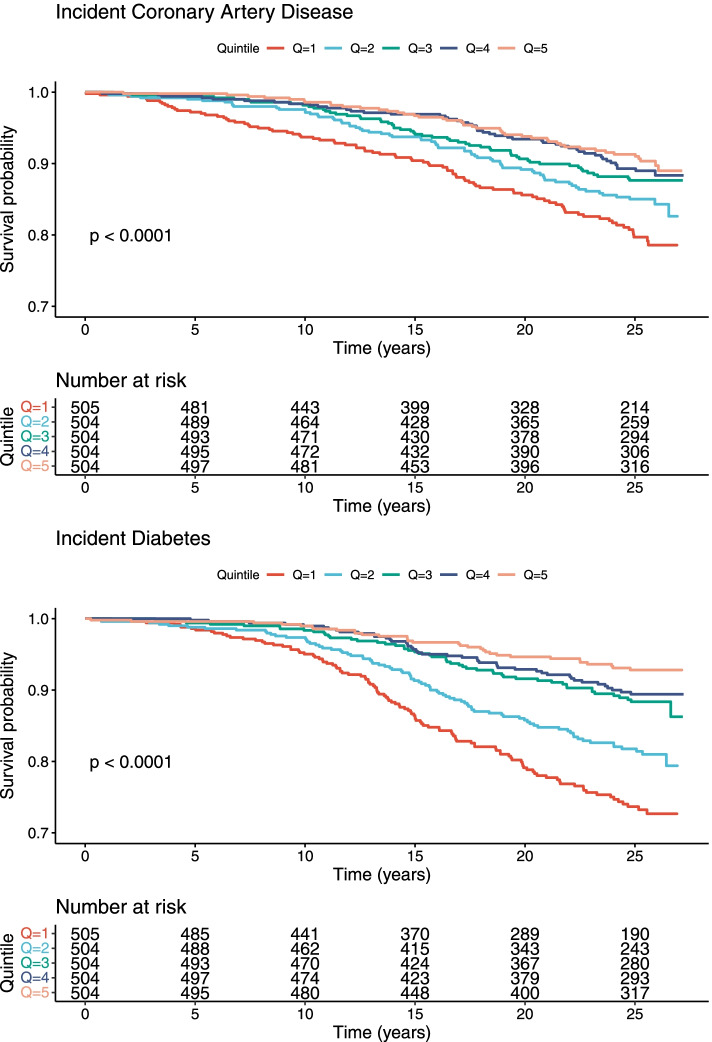
Kaplan–Meier curves. Individuals in MDC split into five quintiles depending on metabolic signature levels. *p*, *p* value calculated using log rank test

In Kaplan–Meier analyses in MDC with participants split into quintiles according to the metabolic signature, lower quintile was associated with an increased risk for both type 2 diabetes and CAD (log rank test *p* < 0.0001) (Fig. 2).

The metabolic signature was associated with a lower risk of type 2 diabetes and CAD in unadjusted models in both MDC and MPP (Table 2). The association with type 2 diabetes was still significant in both MDC (hazard ratio (HR) = 0.73 per 1 SD increment of the metabolic signature, 95% CI 0.63–0.83, *p* = 3E−6) and MPP (odds ratio (OR) = 0.70 per 1 SD increment of the metabolic signature, 95% CI 0.55–0.88, *p* = 0.003) in model 3. The proportional hazard assumption was met for the Cox regression model (Additional file 1: Fig. S2).

**Table 2:** The association between the metabolic signature and future cardiometabolic disease risk in MDC and MPP

**Malmö Diet and Cancer (MDC)**	Model	Person years at risk	*N* cases	Hazard ratio (HR)	*P*
Incident T2D	1	54508	322	0.58 (0.52–0.65)	2E− 22
Incident T2D	2	54508	322	0.58 (0.52–0.66)	3E− 18
Incident T2D	3	54508	322	0.73 (0.63–0.83)	3E− 06
Incident CAD	1	55463	303	0.73 (0.65–0.82)	9E− 08
Incident CAD	2	55463	303	0.87 (0.77–0.99)	0.03
Incident CAD	3	55463	303	0.94 (0.82–1.07)	3
**Malmö Preventive Project (MPP)**	Model	*N* total	*N* cases	Odds ratio (OR)	*p*
Incident T2D	1	701	203	0.53 (0.44–0.63)	2E− 11
Incident T2D	2	701	203	0.54 (0.44–0.65)	1E− 09
Incident T2D	3	701	203	0.70 (0.55–0.88)	0.003
Incident CAD	1	880	382	0.78 (0.68–0.89)	4E− 4
Incident CAD	2	880	382	0.86 (0.74–1.00)	0.06
Incident CAD	3	880	382	0.93 (0.78–1.11)	0.4

Results from Cox proportional hazard models in MDC and logistics regressions in MPP associating the metabolic signature with risk for type 2 diabetes and CAD. Model 2 is adjusted for smoking status, age, sex and physical activity and model 3 is adjusted for smoking status, age, sex, physical activity, LDL cholesterol, HDL cholesterol, glucose, triglycerides, body mass index, systolic blood pressure, and treatment of anti-hypertensive medicine. In MDC, model 2 and 3 was additionally adjusted for alcohol intake

The odds rations and hazard ratios are standardised to 1 SD increment of the metabolic signature

*T2D* Type 2 diabetes, *CAD* Coronary artery disease

The association with CAD remained significant in MDC in model 2 (HR = 0.87 per 1 SD increment of the metabolic signature, 95% CI 0.77–0.99, *p* = 0.03), and in MPP, the association was slightly attenuated (OR = 0.86 per 1 SD increment of the metabolic signature, 95% CI 0.74–1.00, *p* = 0.06) and no longer statistically significant. The Cox regression model for CAD in MDC did not completely fulfil the proportional hazard assumption (Additional file 1: Fig. S3). Associations between the metabolic signature and CAD were thus further analysed with logistic regression, which yielded similar results as the Cox regressions, with model 2 showing no statistically significant association (Additional file 1: Table S6).

The associations with CAD were no longer significant in model 3 in both MDC and MPP (Table 2, Additional file 1: Table S6).

## Discussion

### Key findings

We here identify a metabolite-based signature as a surrogate for a healthy dietary pattern and test its association with future risk for type 2 diabetes and CAD in two independent populations. The metabolic signature was significantly inversely associated with both type 2 diabetes and CAD in two separate cohorts with baseline sampling up to a decade apart. The association between the metabolic signature and type 2 diabetes remained strongly significant after adjustments for several known risk factors.

### Data-driven dietary patterns associated biomarkers

There is an increasing amount of attention given to dietary biomarkers as a tool to assess dietary intake, evaluate compliance to a dietary pattern and to identify and evaluate relationships between dietary patterns and disease [32]. Many studies combining dietary patterns and metabolomics have utilised the methodology to show adherence to pre-determined dietary patterns [12–14, 33, 34]. Several studies have shown that data-driven “prudent” or “health conscious” dietary patterns reflect the highest degree of explained variation in dietary intake [4, 5]. Creating biomarkers for data-driven patterns rather than pre-determined pattern might capture the existing variation in dietary intake in the population. The downside of using data-driven patterns is that external reproducibility is more difficult.

By combining dietary patterns and metabolomics data, it has previously been shown that a Mediterranean diet metabolic signature was associated with a lower risk of cardiovascular disease [35] and metabolites associated with pre-defined healthy dietary indexes have been shown to be associated with a lower risk for type 2 diabetes, albeit the associations were not independent of potential mediators [12]. Prospective cohort studies utilising biomarkers associated to data-driven dietary patterns are scarcer. To our knowledge, a previous publication from our group is the first and only to evaluate data-driven dietary pattern biomarkers association with future disease risk [9]. We discovered metabolites associated with a health conscious dietary pattern and a lower risk for cardiometabolic disease [9]. Here, by using multivariate metabolites modelling, we look to better assess the overall adherence to health conscious dietary patterns as well as the relationship between dietary intake and disease outcome [7]. We also test the relationship between the metabolite modelling and future cardiometabolic disease in a cohort without dietary data to show the potential of such a model.

### Internal and external validation

The cross validation in the training set of MOS and the correlation analysis in the validation set in MOS yielded almost identical results, which indicates that the model was not over-fitted. The moderate correlation between the predicted metabolic signature and the health conscious food pattern in MDC was expected as the food pattern was constructed with a different dietary sampling method, had slightly different loadings, and the plasma was collected more than two decades apart. The metabolic signature in MOS correlated with food groups that were part of the health conscious food pattern [18]. Similarities in the two published health-conscious food patterns in MDC and MOS and the prediction capacity of the metabolic signature suggests that structure of the health-conscious food pattern has remained similar in Sweden over time [6, 18]. It also further supports the case that the metabolic signature reflects healthy eating.

### Dietary metabolites in model loadings

In the model loadings, the top six metabolites all contributed positively. Beta-carotene, ergothioneine and acetylornithine have all been associated with vegetable intake [34, 36, 37]. Homostachydrine (pipecolic acid betaine), is known to be associated with whole grain intake [34] while C4:0:OH-acylcarnitine (hydroxybutyrylcarnitine) has been shown to be associated with fasting in healthy men [38].

The top negative loadings were proline, dimethylguanidino valerate (DMGV) and isoleucine. Rather than specific dietary markers, these metabolites have been shown to represent a state of poor cardiometabolic health associated with an increased risk for type 2 diabetes and CAD [17, 39–41].

### The metabolite signature associates with CAD and type 2 diabetes

The metabolic signature of the healthy dietary pattern was associated with lower risk for CAD and type 2 diabetes in the two separate cohorts MPP and MDC. Individuals with low metabolic signature had a worse risk profile for cardiometabolic disease. However, the association between the metabolic signature and lower risk for type 2 diabetes remained significant in both cohorts even after adjustment for known risk factors. Our model has the potential to identify groups with a higher risk for type 2 diabetes and that increased risk might be due to a poor diet. With further development, similar methods could be used in the future in a clinical setting to assess dietary intake and its contribution to type 2 diabetes risk using a single plasma sample. Here, we calculate the metabolic signature and assess future risk for type 2 diabetes and CAD in cohorts without incorporating dietary data as a proof of concept.

After further adjustment in model 3, the association between the metabolic signature and lower risk for CAD was not significant in neither MPP nor MDC, which implicates that the CAD-association is mediated or confounded by one of the factors in the model, or via an unmeasured factor closely associated with a variable in the model. The addition of dietary based metabolite modelling might provide more insight in type 2 diabetes development than in CAD development.

### Limitations

The reproducibility of our finding are a limitation of the study. We have used two different but similar health conscious food patterns to validate our results, but the structure of such a pattern might be different in other populations. The metabolic signature is also created from an in-house metabolite library that is unique for our lab. To make the results useful in a clinical setting, the biomarker panel could be better optimised for dietary pattern biomarker discovery. The library of metabolites we are measuring focus on amino acids and intermediaries from their degradation pathways. By creating dietary specific biomarkers, perhaps by combining several methods of measurement, prediction of healthy dietary intake using a single plasma sample could be refined.

Another limitation is that metabolite measurements are only conducted once per participants. Repeated plasma sampling could attenuate variation created by the irregular consumption of certain foods. As of now, the application of similar models would be limited to identify groups of individuals with lower adherence to healthy food patterns and higher risk of future cardiometabolic disease and individual assessments should be made with caution.

The nested case-control design in MPP made the application of Cox regression models incorrect. Instead, logistic regression models were used, which might reduce the accuracy of the results slightly due to the time variable not being taken into account. Decreasing the power of the prospective analyses increases the risk of false negative findings.

## Conclusion

In this proof-of-concept study, we identify a metabolic signature as a surrogate for healthy eating that inversely associates with type 2 diabetes independently of a broad set of known risk factors in two independent cohorts. Moreover, the diet-associated metabolic signature was also inversely associated with CAD in both cohorts albeit not independently of known risk factors. We suggest an inverse association between the metabolic signature and cardiometabolic risk and speculate that a lower signature might stem from unhealthy eating habits.

## Supplementary Information

**Additional file 1: Supplementary method.** A more thorough description of the previously published health conscious food patterns as well as more information about the metabolite normalisation process. **Table S1.** A list of measured metabolites and their principal loading in the metabolic signature model. **Figure S1.** A figure with correlations with the dietary pattern, the metabolic signature and food groups in the Malmö Offspring Study. **Table S2.** MOS baseline characteristics by metabolic signature. **Table S3.** MDC baseline characteristics by metabolic signature. **Table S4.** MPP baseline characteristics by metabolic signature. **Table S5.** “One by one” adjustments in MDC. **Figure S2.** Proportionality test for the type 2 diabetes Cox regression. **Figure S3.** Proportionality test for the coronary artery disease Cox regression. **Table S6.** Cox regression compared to logistic regression in MDC.

## Data Availability

The data that support the findings of this study are available from the Malmö Population-Based Cohorts Joint Database but restrictions apply to the availability of these data, which were used under license for the current study, and so are not publicly available. Data are however available from the authors upon reasonable request and with permission of the Malmö Population-Based Cohorts Joint Database. The metabolomics data that support the findings of this study are available from the author upon reasonable request.

## Abbreviations

CAD: Coronary artery disease
MDC: Malmö Diet and Cancer
MOS: Malmö Offspring Study
MPP: Malmö Preventive Project

## References

[1] Vasan RS, Benjamin EJ (2016). The future of cardiovascular epidemiology. Circulation.

[2] Zheng Y, Ley SH, Hu FB (2018). Global aetiology and epidemiology of type 2 diabetes mellitus and its complications. Nat Rev Endocrinol.

[3] Yu E, Malik VS, Hu FB (2018). Cardiovascular disease prevention by diet modification: JACC Health Promotion Series. J Am Coll Cardiol.

[4] McEvoy CT, Cardwell CR, Woodside JV, Young IS, Hunter SJ, McKinley MC (2014). A posteriori dietary patterns are related to risk of type 2 diabetes: findings from a systematic review and meta-analysis. J Acad Nutr Diet.

[5] Rodriguez-Monforte M, Flores-Mateo G, Sanchez E (2015). Dietary patterns and CVD: a systematic review and meta-analysis of observational studies. Br J Nutr.

[6] Ericson U, Brunkwall L, Alves Dias J, Drake I, Hellstrand S, Gullberg B, Sonestedt E, Nilsson PM, Wirfalt E, Orho-Melander M (2019). Food patterns in relation to weight change and incidence of type 2 diabetes, coronary events and stroke in the Malmo Diet and Cancer cohort. Eur J Nutr.

[7] Gao Q, Pratico G, Scalbert A, Vergeres G, Kolehmainen M, Manach C, Brennan L, Afman LA, Wishart DS, Andres-Lacueva C (2017). A scheme for a flexible classification of dietary and health biomarkers. Genes Nutr.

[8] Garcia-Perez I, Posma JM, Gibson R, Chambers ES, Hansen TH, Vestergaard H, Hansen T, Beckmann M, Pedersen O, Elliott P (2017). Objective assessment of dietary patterns by use of metabolic phenotyping: a randomised, controlled, crossover trial. Lancet Diabetes Endocrinol.

[9] Smith E, Ottosson F, Hellstrand S, Ericson U, Orho-Melander M, Fernandez C, Melander O (2020). Ergothioneine is associated with reduced mortality and decreased risk of cardiovascular disease. Heart.

[10] Kim H, Rebholz CM (2021). Metabolomic biomarkers of healthy dietary patterns and cardiovascular outcomes. Curr Atheroscler Rep.

[11] Bar N, Korem T, Weissbrod O, Zeevi D, Rothschild D, Leviatan S, Kosower N, Lotan-Pompan M, Weinberger A, Le Roy CI (2020). A reference map of potential determinants for the human serum metabolome. Nature.

[12] Shi L, Brunius C, Johansson I, Bergdahl IA, Lindahl B, Hanhineva K, Landberg R (2018). Plasma metabolites associated with healthy Nordic dietary indexes and risk of type 2 diabetes-a nested case-control study in a Swedish population. Am J Clin Nutr.

[13] McCullough ML, Maliniak ML, Stevens VL, Carter BD, Hodge RA, Wang Y (2019). Metabolomic markers of healthy dietary patterns in US postmenopausal women. Am J Clin Nutr.

[14] Rebholz CM, Lichtenstein AH, Zheng Z, Appel LJ, Coresh J (2018). Serum untargeted metabolomic profile of the Dietary Approaches to Stop Hypertension (DASH) dietary pattern. Am J Clin Nutr.

[15] Brunkwall L, Jonsson D, Ericson U, Hellstrand S, Kennback C, Ostling G, Jujic A, Melander O, Engstrom G, Nilsson J (2021). The Malmo Offspring Study (MOS): design, methods and first results. Eur J Epidemiol.

[16] Berglund G, Elmstahl S, Janzon L, Larsson SA (1993). The Malmo diet and cancer study. Design and feasibility. J Intern Med.

[17] Ottosson F, Smith E, Melander O, Fernandez C (2018). Altered asparagine and glutamate homeostasis precede coronary artery disease and type 2 diabetes. J Clin Endocrinol Metab.

[18] Ericson U, Brunkwall L, Hellstrand S, Nilsson PM, Orho-Melander M (2020). A health-conscious food pattern is associated with prediabetes and gut microbiota in the Malmo Offspring Study. J Nutr.

[19] Tremmel M, Lyssenko V, Zöller B, Engström G, Magnusson M, Melander O, Nilsson PM, Bachus E (2018). Characteristics and prognosis of healthy severe obesity (HSO) subjects - The Malmo Preventive Project. Obes Med.

[20] Ludvigsson JF, Andersson E, Ekbom A, Feychting M, Kim JL, Reuterwall C, Heurgren M, Olausson PO (2011). External review and validation of the Swedish national inpatient register. BMC Public Health.

[21] Lagerqvist B, James SK, Stenestrand U, Lindback J, Nilsson T, Wallentin L, Group SS (2007). Long-term outcomes with drug-eluting stents versus bare-metal stents in Sweden. N Engl J Med.

[22] Enhorning S, Sjogren M, Hedblad B, Nilsson PM, Struck J, Melander O (2016). Genetic vasopressin 1b receptor variance in overweight and diabetes mellitus. Eur J Endocrinol.

[23] Elmstahl S, Gullberg B, Riboli E, Saracci R, Lindgarde F (1996). The Malmo Food Study: the reproducibility of a novel diet history method and an extensive food frequency questionnaire. Eur J Clin Nutr.

[24] Callmer E, Riboli E, Saracci R, Akesson B, Lindgarde F (1993). Dietary assessment methods evaluated in the Malmo food study. J Intern Med.

[25] Nybacka S, Berteus Forslund H, Wirfalt E, Larsson I, Ericson U, Warensjo Lemming E, Bergstrom G, Hedblad B, Winkvist A, Lindroos AK (2016). Comparison of a web-based food record tool and a food-frequency questionnaire and objective validation using the doubly labelled water technique in a Swedish middle-aged population. J Nutr Sci.

[26] Hellstrand S, Ottosson F, Smith E, Brunkwall L, Ramne S, Sonestedt E, Nilsson PM, Melander O, Orho-Melander M, Ericson U (2021). Dietary data in the Malmö Offspring Study-reproducibility, method comparison and validation against objective biomarkers. Nutrients.

[27] Ottosson F, Ericson U, Almgren P, Nilsson J, Magnusson M, Fernandez C, Melander O (2016). Postprandial levels of branch chained and aromatic amino acids associate with fasting glycaemia. J Amino Acids.

[28] Dunn WB, Broadhurst D, Begley P, Zelena E, Francis-McIntyre S, Anderson N, Brown M, Knowles JD, Halsall A, Haselden JN (2011). Procedures for large-scale metabolic profiling of serum and plasma using gas chromatography and liquid chromatography coupled to mass spectrometry. Nat Protoc.

[29] Rohart F, Gautier B, Singh A, Le Cao KA (2017). mixOmics: an R package for ‘omics feature selection and multiple data integration. PLoS Comput Biol.

[30] Lê Cao KA, Rossouw D, Robert-Granié C, Besse P (2008). A sparse PLS for variable selection when integrating omics data. Stat Appl Genet Mol Biol.

[31] Therneau T (2021). A package for survival analysis in R. R package version 3.2-13 ed.

[32] Brennan L, Hu FB (2019). Metabolomics-based dietary biomarkers in nutritional epidemiology-current status and future opportunities. Mol Nutr Food Res.

[33] Walker ME, Song RJ, Xu X, Gerszten RE, Ngo D, Clish CB, Corlin L, Ma J, Xanthakis V, Jacques PF (2020). Proteomic and metabolomic correlates of healthy dietary patterns: the Framingham Heart Study. Nutrients.

[34] Playdon MC, Moore SC, Derkach A, Reedy J, Subar AF, Sampson JN, Albanes D, Gu F, Kontto J, Lassale C (2017). Identifying biomarkers of dietary patterns by using metabolomics. Am J Clin Nutr.

[35] Li J, Guasch-Ferre M, Chung W, Ruiz-Canela M, Toledo E, Corella D, Bhupathiraju SN, Tobias DK, Tabung FK, Hu J (2020). The Mediterranean diet, plasma metabolome, and cardiovascular disease risk. Eur Heart J.

[36] Scalbert A, Brennan L, Manach C, Andres-Lacueva C, Dragsted LO, Draper J, Rappaport SM, van der Hooft JJ, Wishart DS (2014). The food metabolome: a window over dietary exposure. Am J Clin Nutr.

[37] Pallister T, Jennings A, Mohney RP, Yarand D, Mangino M, Cassidy A, MacGregor A, Spector TD, Menni C (2016). Characterizing blood metabolomics profiles associated with self-reported food intakes in female twins. PLoS One.

[38] Soeters MR, Serlie MJ, Sauerwein HP, Duran M, Ruiter JP, Kulik W, Ackermans MT, Minkler PE, Hoppel CL, Wanders RJ (2012). Characterization of D-3-hydroxybutyrylcarnitine (ketocarnitine): an identified ketosis-induced metabolite. Metabolism.

[39] Wang TJ, Larson MG, Vasan RS, Cheng S, Rhee EP, McCabe E, Lewis GD, Fox CS, Jacques PF, Fernandez C (2011). Metabolite profiles and the risk of developing diabetes. Nat Med.

[40] Ottosson F, Ericson U, Almgren P, Smith E, Brunkwall L, Hellstrand S, Nilsson PM, Orho-Melander M, Fernandez C, Melander O (2019). Dimethylguanidino valerate: a lifestyle-related metabolite associated with future coronary artery disease and cardiovascular mortality. J Am Heart Assoc.

[41] Magnusson M, Lewis GD, Ericson U, Orho-Melander M, Hedblad B, Engstrom G, Ostling G, Clish C, Wang TJ, Gerszten RE (2013). A diabetes-predictive amino acid score and future cardiovascular disease. Eur Heart J.

