# Supplementary material for: A healthy dietary metabolic signature is associated with a lower risk for type 2 diabetes and coronary artery disease
Source: BMC Med. 2022 Apr 21;20:122. doi: 10.1186/s12916-022-02326-z (PMC9022292; doi:10.1186/s12916-022-02326-z)
Supplement: Supplementary file 1 — Additional file 1: Supplementary method. A more thorough description of the previously published health conscious food patterns as well as more information about the metabolite normalisation process. Table S1. A list of measured metabolites and their principal loading in the metabolic signature model. Figure S1. A figure with correlations with the dietary pattern, the metabolic signature and food groups in the Malmö Offspring Study. Table S2. MOS baseline characteristics by metabolic signature. Table S3. MDC baseline characteristics by metabolic signature. Table S4. MPP baseline characteristics by metabolic signature. Table S5. “One by one” adjustments in MDC. Figure S2. Proportionality test for the type 2 diabetes Cox regression. Figure S3. Proportionality test for the coronary artery disease Cox regression. Table S6. Cox regression compared to logistic regression in MDC. [file 12916_2022_2326_MOESM1_ESM.docx]

Additional file 1 for

A healthy dietary metabolic signature is associated with a lower risk for type 2 diabetes and coronary artery disease

Einar Smith MD^1^, Ulrika Ericson PhD^1^, Sophie Hellstrand PhD^1^, Marju Orho-Melander PhD^1^, Peter M Nilsson MD PhD^1,2^, Céline Fernandez^1^, Olle Melander MD PhD^1,2^, Filip Ottosson PhD^1,3.^

^1^Department of Clinical Sciences, Lund University, Malmö, Sweden

^2^Department of Emergency and Internal Medicine, Skåne University Hospital, Malmö, Sweden
^3^Section for Clinical Mass Spectrometry, Danish Center for Neonatal Screening, Department of Congenital Disorders, Statens Serum Institut, Copenhagen, Denmark

**Supplementary method**

**Structure of the health conscious food patterns**

The health-conscious food pattern in MOS was characterized by high intakes of fruits and berries, nuts and seeds, legumes, other vegetables, plain yoghurt, fresh cheese, tea, animal replacements foods, breakfast cereals, cooked grains, oil-based dressings, fish and fibre-rich bread and by low intakes of sugar-sweetened beverages, red and processed meat, white bread and fried/deep-fried potatoes (1). The health-conscious food pattern in MDC was created separately in men and women but due to their similarity, they are merged in this project (2). The food pattern was characterized by high intakes of fibre-rich bread, fruits, vegetables, breakfast cereals, fish and low-fat yoghurt, and by low intake of low-fibre bread. In men, a high intake of cream and in women cottage cheese was also characterising the pattern. Rather low intakes of red and processed meat and sugar-sweetened beverages could also be noted in both genders. (2). The complete scoring of individual food groups in relations to the health conscious dietary patterns are available in the original publications on MOS and MDC (1, 2).

**Metabolite normalisation**

Quality control samples were injected in the beginning and after every eight analytical samples in order to ensure high analytical repeatability. All metabolites were normalized to standard curves calculated from the quality control samples. Briefly, a low-order nonlinear locally estimated smoothing function was fitted to the signals from each metabolite in the quality control samples as a function of the injection order. Using this function, correction curves for each metabolites’ analytical samples were interpolated, to which the metabolite measurements in the analytical samples were normalized (3). Metabolites signals were then mean centred and unit-variance scaled. Outliers were defined as values differing 5 standard deviations from the mean. Low outliers were imputed to the lowest value detected and high outlier were imputed to +5 standard deviations. Out of all values, 1.79% were imputed.

**Table S1:** Metabolite library

| Metabolite | Loading  component 1 | HMDBID |
| --- | --- | --- |
| Beta-carotene | 0.36 | HMDB00561 |
| Acylcarnitine C4:0-OH | 0.24 | Putative: HMDB13127 |
| Ergothioneine | 0.23 | HMDB03045 |
| Homostachydrine | 0.21 | HMDB33433 |
| Acylcarnitine C13:0 | 0.21 | Putative: HMDB0241308 |
| Acetylornithine | 0.20 | HMDB03357 |
| Proline | -0.20 | HMDB00162 |
| DMGV | -0.18 | HMDB0240212 |
| Hippurate | 0.17 | HMDB00714 |
| Isoleucine | -0.17 | HMDB00172 |
| Leucine | -0.16 | HMDB00687 |
| 4-Trimethylammoniobutanoic acid | -0.15 | HMDB01161 |
| Proline betaine | 0.15 | HMDB04827 |
| Urobilin | -0.15 | HMDB04160 |
| Creatinine | -0.14 | HMDB00562 |
| Phenylacetylglutamine | 0.13 | HMDB06344 |
| 25-Hydroxyvitamin D3 | -0.13 | Putative: HMDB03550 |
| Acylcarnitine C13:1 | 0.13 | Putative |
| Acylcarnitine C11:0 | 0.12 | Putative: HMDB13321 |
| Acylcarnitine C8:1 | -0.12 | Putiative: Putative |
| Acylcarnitine C10:3 | -0.12 | Putative |
| Glutamate | -0.11 | HMDB00148 |
| Trigonelline | 0.11 | HMDB00875 |
| Pipecolate | 0.11 | HMDB00716 |
| Cotinine | -0.11 | HMDB01046 |
| Dimethylglycine | -0.10 | HMDB00092 |
| Carnitine | -0.10 | HMDB00062 |
| Tyrosine | 0.10 | HMDB00158 |
| 1-Methylhistidine | -0.10 | HMDB00001 |
| 3-Hydroxytrimethyllysine | -0.10 | HMDB01422 |
| Trimethyllysine | -0.10 | HMDB01325 |
| L-NMMA | 0.10 | HMDB29416 |
| Acylcarnitine C14:2 | 0.09 | Putative: HMDB13331 |
| Methionine-S-oxide | 0.09 | HMDB02005 |
| Acylcarnitine C10:1 | 0.09 | Putative: HMDB13205 |
| 7-Methylguanine | -0.09 | HMDB00897 |
| Acylcarnitine C9:0 | 0.09 | Putative: HMDB13288 |
| Hydroxycotinine | -0.09 | HMDB01390 |
| N2.N2.Dimethylguanosine | -0.08 | HMDB04824 |
| Kynurenate | -0.08 | HMDB00715 |
| Tiglylcarnitine | 0.08 | Putative: HMDB02366 |
| Acylcarnitine C3:0 | -0.08 | HMDB00824 |
| N-Methylproline | 0.07 | HMDB94696 |
| Acylcarnitine C12:2 | 0.07 | Putative |
| Acylcarnitine C12:0 | 0.07 | Putative: HMDB02250 |
| Lysine | -0.07 | HMDB00182 |
| Homocitrulline | 0.06 | HMDB00679 |
| Acylcarnitine C10:0 | 0.06 | Putative: HMDB00651 |
| Acylcarnitine C14:0 | 0.06 | HMDB05066 |
| Tryptophan | -0.06 | HMDB00929 |
| Acylcarnitine C5:0 | -0.06 | HMDB00688 |
| Asparagine | 0.06 | HMDB00168 |
| Glutamine | -0.05 | HMDB00641 |
| Acisoga | 0.05 | HMDB61384 |
| Threonine | -0.05 | HMDB00167 |
| Kynurenine | -0.05 | HMDB00684 |
| Acylcarnitine C10:0-OH | 0.05 | Putative |
| Methyllysine | -0.05 | HMDB02038 |
| Valine | -0.05 | HMDB00883 |
| Acylcarnitine C12:1 | 0.05 | Putative: HMDB13326 |
| Nicotinamide | -0.05 | HMDB01406 |
| Pyroglutamate | -0.05 | HMDB00267 |
| Homoarginine | -0.04 | HMDB00670 |
| Urocanate | -0.04 | HMDB00301 |
| N-Methyl-4-pyridone-3-carboxamide | -0.04 | HMDB04194 |
| Acylcarnitine C8:0 | 0.04 | HMDB00791 |
| Piperine | -0.04 | HMDB29377 |
| Paraxanthine | 0.04 | HMDB01860 |
| Acylcarnitine C16:1 | 0.04 | Putative: HMDB13207 |
| 5-Acetylamino-6-amino-3-methyluracil | 0.04 | HMDB04400 |
| Urea | -0.04 | HMDB00294 |
| Methionine | 0.04 | HMDB00696 |
| Acylcarnitine C8:0-OH | 0.04 | Putative |
| Guanidineacetate | -0.04 | HMDB00128 |
| Serine | 0.03 | HMDB00187 |
| Hypoxanthine | -0.03 | HMDB00157 |
| Acetylcarnosine | -0.03 | HMDB12881 |
| Pantothenate | 0.03 | HMDB00210 |
| Arginine | -0.03 | HMDB00517 |
| Phenylalanine | -0.03 | HMDB00159 |
| Acylcarnitine C18:1 | -0.03 | Putative |
| Acylcarnitine C14:1 | 0.03 | Putative: HMDB0240588 |
| Cystine | -0.03 | HMDB00192 |
| Trimethylamine-N-oxide | 0.02 | HMDB00925 |
| Acylcarnitine C2:0 | 0.02 | HMDB00201 |
| Alanine | -0.02 | HMDB00161 |
| Acylcarnitine C16:0 | -0.02 | HMDB00222 |
| Histidine | -0.02 | HMDB00177 |
| Dimethyllysine | -0.02 | Putative: HMDB13287 |
| 1-Methyladenosine | 0.02 | HMDB03331 |
| Acylcarnitine C11:1 | 0.02 | Putative |
| Choline | 0.02 | HMDB00097 |
| Citrulline | 0.02 | HMDB00904 |
| Acetylarginine | -0.02 | HMDB04620 |
| Betaine | 0.02 | HMDB00043 |
| Acylcarnitine C4:0 | 0.01 | HMDB02013 |
| Asymmetric dimethylarginine | 0.01 | HMDB01539 |
| Methylnicotinamide | -0.01 | HMDB03152 |
| Caffeine | 0.01 | HMDB01847 |
| Glycerophosphocholine | -0.01 | HMDB00086 |
| 5-Methylthioadenosine | 0.005 | HMDB01173 |
| 3-Methylhistidine | 0.004 | HMDB00479 |
| Acylcarnitine C18:2 | -0.003 | Putative |
| Acylcarnitine C10:2 | -0.003 | Putative |
| Acylcarnitine C6:0 | 0.003 | Putative: HMDB00756 |
| Symmetric dimethylarginine | 0.003 | HMDB03334 |
| 2-Aminoisobutyric acid | 0.002 | HMDB01906 |
| Taurine | -0.002 | HMDB00251 |
| Acylcarnitine C18:0 | -0.001 | HMDB00848 |
| Creatine | -0.001 | HMDB00064 |
| Ornithine | -0.001 | HMDB00214 |

*Overlap of metabolites measured in Malmö Offspring Study, Malmö diet and Cancer and Malmö Preventive project. Loadings presented are the loadings from the single component in the partial least square regression model created in MOS. Metabolites with putative ID has been matched via fragmentation spectra, the remaining metabolites have identities confirmed by comparison with a standard.*

**Figure S1**: Metabolite signature and association with food groups*.
Pearson correlations between the dietary pattern, the metabolic signature and intake of food groups in the Malmö offspring study. Diet score: The health conscious food pattern in MOS. SSB: Sugar sweetened beverages*

***Table S2:*** MOS baseline characteristics by metabolic signature

*Baseline* *characteristics summaries by quintile of the metabolic signature. P-value is calculated using ANOVA for continuous variables and chi-square test for categorical variables. Adjusted p-value is adjusted for sex and age. SBP: Systolic blood pressure.*

| MOS |  | Q1 (N=308) | Q2 (N=307) | Q3 (N=308) | Q4 (N=307) | Q5 (N=308) | Total (N=1538) | p value | Adjusted p-value |
| --- | --- | --- | --- | --- | --- | --- | --- | --- | --- |
| Age (years) | Mean (SD) | 37.6 (13.6) | 38.5 (13.7) | 38.7 (13.7) | 41.3 (13.7) | 44.0 (13.4) | 40.0 (13.8) | < 0.001 | < 0.001 |
| Female |  | 60 (19.5%) | 142 (46.3%) | 173 (56.2%) | 218 (71.0%) | 244 (79.2%) | 837 (54.4%) | < 0.001 | < 0.001 |
| Physical activity | 1 | 175 (62.1%) | 177 (62.5%) | 183 (65.1%) | 176 (60.5%) | 189 (65.6%) | 900 (63.2%) | 0.005 | 0.3 |
|  | 2 | 17 (6.0%) | 22 (7.8%) | 10 (3.6%) | 12 (4.1%) | 5 (1.7%) | 66 (4.6%) |  |  |
|  | 3 | 31 (11.0%) | 27 (9.5%) | 26 (9.3%) | 26 (8.9%) | 13 (4.5%) | 123 (8.6%) |  |  |
|  | 4 | 59 (20.9%) | 57 (20.1%) | 62 (22.1%) | 77 (26.5%) | 81 (28.1%) | 336 (23.6%) |  |  |
| Smokers |  | 48 (17.0%) | 49 (17.3%) | 36 (12.8%) | 38 (13.1%) | 18 (6.2%) | 189 (13.3%) | < 0.001 | < 0.001 |
| LDL  (mmol L^-1^) | Mean (SD) | 3.3 (1.0) | 3.1 (0.9) | 3.1 (0.9) | 3.1 (0.9) | 3.1 (0.9) | 3.1 (0.9) | 0.06 | 0.02 |
| HDL  (mmol L^-1^) | Mean (SD) | 1.3 (0.3) | 1.5 (0.3) | 1.7 (0.4) | 1.8 (0.4) | 2.0 (0.5) | 1.7 (0.5) | < 0.001 | < 0.001 |
| TG  (mmol L^-1^) | Mean (SD) | 1.5 (0.9) | 1.2 (0.6) | 1.0 (0.5) | 0.9 (0.4) | 0.8 (0.3) | 1.1 (0.6) | < 0.001 | < 0.001 |
| Glucose (mmol L^-1^) | Mean (SD) | 5.4 (0.7) | 5.4 (0.7) | 5.3 (0.6) | 5.4 (0.7) | 5.3 (0.5) | 5.4 (0.6) | 0.09 | 0.08 |
| BMI  (kg m^-2^) | Mean (SD) | 27.9 (5.2) | 26.3 (4.5) | 25.1 (4.0) | 24.4 (3.8) | 23.6 (3.0) | 25.4 (4.4) | < 0.001 | < 0.001 |
| SBP  (mm Hg) | Mean (SD) | 120.2 (14.3) | 118.9 (14.7) | 116.2 (14.8) | 115.2 (16.9) | 115.6 (15.5) | 117.2 (15.4) | < 0.001 | < 0.001 |
| Anti-hypertensive medicine |  | 25 (9.2%) | 25 (9.1%) | 21 (7.7%) | 17 (5.9%) | 17 (5.9%) | 105 (7.5%) | 0.4 | 0.3 |

***Table S3:*** MDC baseline characteristics by metabolic signature*.*

| MDC |  | Q1 (N=505) | Q2 (N=504) | Q3 (N=504) | Q4 (N=504) | Q5 (N=504) | Total (N=2521) | p value | Adjusted p-value |
| --- | --- | --- | --- | --- | --- | --- | --- | --- | --- |
| Age (years) | Mean (SD) | 57.4  (6.3) | 57.4 (6.0) | 57.1 (5.9) | 57.3  (6.0) | 57.8 (5.9) | 57.4  (6.0) | 0.5 | 0.5 |
| Female |  | 163 (32.4%) | 261 (51.6%) | 295  (58.6%) | 371 (73.5%) | 411 (81.6%) | 1501 (59.5%) | < 0.001 | < 0.001 |
| Physical activity | Low | 207 (41.0%) | 164 (32.5%) | 169 (33.5%) | 176 (34.9%) | 125 (24.8%) | 841 (33.4%) | < 0.001 | < 0.001 |
|  | Medium | 166 (32.9%) | 161 (31.9%) | 178 (35.3%) | 166 (33.9%) | 169 (33.5%) | 840 (33.3%) |  |  |
|  | High | 132 (26.1%) | 179  (35.5%) | 157 (31.2%) | 162 (32.1%) | 210 (41.7%) | 840 (33.3%) |  |  |
| Current  Smokers | % | 170 (33.7%) | 173 (34.3%) | 134 (26.6%) | 137 (27.2%) | 95 (18.8%) | 709 (28.1%) | < 0.001 | < 0.001 |
| LDL  (mmol L^-1^) | Mean (SD) | 4.0  (0.9) | 4.2  (1.0) | 4.1  (1.0) | 4.1  (1.0) | 4.1  (1.0) | 4.1  (1.0) | 0.06 | 0.05 |
| HDL  (mmol L^-1^) | Mean (SD) | 1.2  (0.3) | 1.3  (0.3) | 1.4  (0.3) | 1.5  (0.3) | 1.7  (0.4) | 1.4  (0.4) | < 0.001 | < 0.001 |
| TG  (mmol L^-1^) | Mean (SD) | 1.5  (0.7) | 1.3  (0.6) | 1.2  (0.5) | 1.1  (0.5) | 1.0  (0.4) | 1.2  (0.6) | < 0.001 | < 0.001 |
| Glucose (mmol L^-1^) | Mean (SD) | 5.0  (0.4) | 5.0  (0.4) | 4.9  (0.4) | 4.9  (0.4) | 4.8  (0.4) | 4.9  (0.4) | < 0.001 | < 0.001 |
| BMI  (kg m^-2^) | Mean (SD) | 26.8  (4.1) | 26.0 (3.7) | 25.1 (3.5) | 24.6 (3.2) | 23.8 (2.9) | 25.3  (3.7) | < 0.001 | < 0.001 |
| SBP  (mm Hg) | Mean (SD) | 143.4 (18.8) | 140.7 (18.1) | 141.1 (18.7) | 139.0 (18.0) | 138.8 (18.6) | 140.6 (18.5) | < 0.001 | < 0.001 |
| Anti-hypertensive medicine |  | 80 (15.8%) | 55 (10.9%) | 54 (10.7%) | 52 (10.3%) | 43 (8.5%) | 284 (11.3%) | 0.006 | 0.006 |
| Alcohol intake (g day) | Mean (SD) | 11.1 (13.8) | 10.3 (11.2) | 10.8 (12.0) | 10.9 (12.3) | 9.6  (9.5) | 10.5 (11.9) | 0.2 | 0.2 |

*Baseline characteristics summaries by quintile of the metabolic signature. P-value is calculated using ANOVA for continuous variables and chi-square test for categorical variables. Adjusted p-value is adjusted for sex and age. SBP: Systolic blood pressure*

| MPP |  | Q1 (N=210) | Q2 (N=210) | Q3 (N=210) | Q4 (N=210) | Q5 (N=210) | Total (N=1049) | p value | Adjusted p |
| --- | --- | --- | --- | --- | --- | --- | --- | --- | --- |
| Age (years) | Mean (SD) | 68.7  (5.9) | 69.5  (6.0) | 68.7  (5.8) | 70.5  (6.0) | 70.2  (6.5) | 69.5  (6.1) | 0.003 | 0.002 |
| Female |  | 39 (13.0%) | 65 (21.7%) | 75 (25.0%) | 108 (36.0%) | 142 (47.3%) | 429 (28.6%) | < 0.001 | < 0.001 |
| Physical activity | Low | 32 (15.2%) | 41 (19.5%) | 24 (11.5%) | 21 (10.0%) | 12 (5.7%) | 130 (12.4%) | < 0.001 | 0.04 |
|  | Medium | 160 (76.2%) | 147 (70.0%) | 154 (73.7%) | 153 (72.9%) | 157 (74.8%) | 771 (73.5%) |  |  |
|  | High | 18  (8.6%) | 22 (10.5%) | 31 (14.8%) | 36 (17.1%) | 41 (19.5%) | 148 (14.1%) |  |  |
| Current smokers |  | 57 (27.1%) | 57 (27.1%) | 49 (23.4%) | 33 (15.7%) | 29 (13.8%) | 225 (21.4%) | < 0.001 | < 0.001 |
| LDL  (mmol L^-1^) | Mean (SD) | 3.7  (1.0) | 3.8  (0.9) | 3.7  (1.0) | 3.9  (1.0) | 3.8  (1.0) | 3.8  (1.0) | 0.06 | 0.3 |
| HDL  (mmol L^-1^) | Mean (SD) | 1.1  (0.3) | 1.3  (0.3) | 1.3  (0.4) | 1.5  (0.4) | 1.6  (0.4) | 1.4  (0.4) | < 0.001 | < 0.001 |
| TG  (mmol L^-1^) | Mean (SD) | 1.6 (0.7) | 1.3 (0.6) | 1.2 (0.5) | 1.1 (0.6) | 1.0 (0.5) | 1.2  (0.6) | < 0.001 | < 0.001 |
| Glucose (mmol L^-1^) | Mean (SD) | 5.7  (0.6) | 5.6  (0.5) | 5.6  (0.6) | 5.5  (0.6) | 5.3  (0.5) | 5.5  (0.6) | < 0.001 | < 0.001 |
| BMI  (kg m^-2^) | Mean (SD) | 29.0  (4.3) | 28.2  (4.6) | 27.4  (4.1) | 26.1  (3.8) | 25.0  (3.6) | 27.1  (4.3) | < 0.001 | < 0.001 |
| SBP  (mm Hg) | Mean (SD) | 146.3 (20.6) | 148.4 (23.0) | 145.4 (22.7) | 147.5 (21.3) | 144.2 (19.5) | 146.4 (21.5) | 0.3 | 0.3 |
| Anti-hypertensive medicine |  | 82 (39.0%) | 76 (36.2%) | 53 (25.4%) | 67 (31.9%) | 68 (32.4%) | 346 (33.0%) | 0.04 | 0.004 |

***Table S4:*** MPP baseline characteristics by metabolic signature

*Baseline* *characteristics summaries by quintile of the metabolic signature. P-value is calculated using ANOVA for continuous variables and chi-square test for categorical variables. Adjusted p-value is adjusted for sex and age. SBP: Systolic blood pressure.*

***Table S5:*** Cox regression analyses associating the metabolic signature with incident type 2 diabetes in Malmö Diet and Cancer (MDC).

|  | Hazard Ratio (HR) | p-value |
| --- | --- | --- |
| Model 1 | 0.58 (0.52-0.65) | 2E-22 |
| Model 2 (ag e+ gender + smoking status + Physcial activity + alcohol intake) | 0.58 (0.52-0.66) | 2E-18 |
| Model 2 + LDL cholesterol | 0.58 (0.51, 0.65) | 1E-18 |
| Model 2 + HDL cholesterol | 0.63 (0.55-0.71) | 1E-12 |
| Model 2 + BMI | 0.73 (0.59-0.76) | 7E-10 |
| Model 2 + fasting glucose | 0.63 (0.56-0.72) | 2E-12 |
| Model 2 + systolic blood pressure | 0.60 (0.53-0.67) | 1E-16 |
| Model 2 + antihypertensive treatment | 0.59 (0.52-0.67) | 3E-17 |
| Model 3 (Model 2 + LDL+HDL+BMI+Glucose+ systolic blood pressure + antihypertensive treatment) | 0.73 (0.63-0.83) | 3E-6 |

*The hazard ratios are standardized to 1 SD increment of the metabolic signature, LDL: LDL*

******

**Figure S2*:*** Proportionality test for type 2 diabetes Cox regression. *Scaled Schoenfeld residuals along with a smooth curve with 95% confidence interval. MKC_signature: The metabolic signature. SBP: Systolic blood pressure, AHT: Antihypertensive treatment. PA: Physical activity.*

******

**Figure S3*:*** Proportionality test for coronary artery disease Cox regression. *Scaled Schoenfeld residuals along with a smooth curve with 95% confidence interval. MKC_signature: The metabolic signature. SBP: Systolic blood pressure, AHT: Antihypertensive treatment. PA: Physical activity.*

**Table S6:** Cox regression compared to logistic regressions in MDC. *Model 1 is unadjusted. Model 2 is adjusted for smoking status, age, sex, alcohol intake and physical activity and model 3 is adjusted for smoking status, age, sex, alcohol intake, physical activity, LDL cholesterol, HDL cholesterol, glucose, triglycerides, body mass index, systolic blood pressure, and treatment of anti-hypertensive medicine. CAD: Coronary artery disease, Cox = Cox proportional hazard regression. Log = Logistic regression*

| **Malmö Diet and Cancer (MDC)** | Hazard Ratio (HR) or Odds ratio (OR) | p | N cases | N total | Model | Method |
| --- | --- | --- | --- | --- | --- | --- |
| Incident CAD | 0.73 (0.65-0.82) | 9E-08 | 303 | 2521 | 1 | Cox |
| Incident CAD | 0.87 (0.77-0.99) | 0.03 | 303 | 2521 | 2 | Cox |
| Incident CAD | 0.94 (0.82-1.07) | 0.3 | 303 | 2521 | 3 | Cox |
| Incident CAD | 0.75 (0.67-0.85) | 4E-6 | 303 | 2521 | 1 | Log |
| Incident CAD | 0.89 (0.78-1.01) | 0.08 | 303 | 2521 | 2 | Log |
| Incident CAD | 0.96 (0.83-1.11) | 0.6 | 303 | 2521 | 3 | Log |

**Supplementary material references**

1. Ericson U, Brunkwall L, Hellstrand S, Nilsson PM, Orho-Melander M. A Health-Conscious Food Pattern Is Associated with Prediabetes and Gut Microbiota in the Malmo Offspring Study. J Nutr. 2020;150:861-72.

2. Ericson U, Brunkwall L, Alves Dias J, Drake I, Hellstrand S, Gullberg B, Sonestedt E, Nilsson PM, Wirfalt E, Orho-Melander M. Food patterns in relation to weight change and incidence of type 2 diabetes, coronary events and stroke in the Malmo Diet and Cancer cohort. Eur J Nutr. 2019;58:1801-14.

3. Dunn WB, Broadhurst D, Begley P, Zelena E, Francis-McIntyre S, Anderson N, Brown M, Knowles JD, Halsall A, Haselden JN, et al. Procedures for large-scale metabolic profiling of serum and plasma using gas chromatography and liquid chromatography coupled to mass spectrometry. Nat Protoc. 2011;6:1060-83.
